# On coughing and airborne droplet transmission to humans

**DOI:** 10.1063/5.0011960

**Published:** 2020-05-01

**Authors:** Talib Dbouk, Dimitris Drikakis

**Affiliations:** University of Nicosia, Nicosia CY-2417, Cyprus

## Abstract

Our understanding of the mechanisms of airborne transmission of viruses is incomplete. This paper employs computational multiphase fluid dynamics and heat transfer to investigate transport, dispersion, and evaporation of saliva particles arising from a human cough. An ejection process of saliva droplets in air was applied to mimic the real event of a human cough. We employ an advanced three-dimensional model based on fully coupled Eulerian–Lagrangian techniques that take into account the relative humidity, turbulent dispersion forces, droplet phase-change, evaporation, and breakup in addition to the droplet–droplet and droplet–air interactions. We computationally investigate the effect of wind speed on social distancing. For a mild human cough in air at 20 °C and 50% relative humidity, we found that human saliva-disease-carrier droplets may travel up to unexpected considerable distances depending on the wind speed. When the wind speed was approximately zero, the saliva droplets did not travel 2 m, which is within the social distancing recommendations. However, at wind speeds varying from 4 km/h to 15 km/h, we found that the saliva droplets can travel up to 6 m with a decrease in the concentration and liquid droplet size in the wind direction. Our findings imply that considering the environmental conditions, the 2 m social distance may not be sufficient. Further research is required to quantify the influence of parameters such as the environment’s relative humidity and temperature among others.

## I. INTRODUCTION

The recent COVID-19 pandemic prompted the need for deeper understanding of the transport of fluids and particles emanating from our respiratory tracts when we cough, sneeze, speak, or breathe. The particles’ transport will influence the spread of coronavirus and determine the implementation of guidelines on social distancing, mask wearing, crowded gatherings, as well as everyday practices of social behavior in private, public, and business environments.

When sneezing or coughing, larger droplets are formed by saliva and smaller droplets by the mucous coating of the lungs and vocal cords. The smaller droplets are often invisible to the naked eye. Past research has shown that most respiratory droplets do not travel independently on their trajectories. Instead, droplets in a continuum of sizes are trapped and carried forward within a moist, warm, turbulent cloud of gas.^1^ In another study, it was shown that as people raise their voice, they emit more droplets, but the size distribution of the droplets remains the same.^2^ Furthermore, researchers have shown that even breathing could release potentially infectious aerosols.^3^ They have captured the large droplets produced when sneezing and coughing as well as the aerosol droplets produced when sneezing, coughing, breathing, and talking on different surfaces. Yan *et al.*^3^ showed that the flu virus exists even in the tiny droplets resulting from breathing or talking alone. Although the mechanisms of transmission are still under debate, it is widely accepted that aerosol or respiratory droplet transmission is the critical factor for the rapid spread and continued circulation of influenza A virus in humans.^4^

The National Academies Standing Committee on Emerging Infectious Diseases and 21st Century Health Threats has considered whether the SARS-CoV-2 virus could be spread through conversation, in addition to the transmission due to sneeze/cough droplets.^5^ As Beans^6^ reported, the team determined that the current evidence supports the possibility that SARS-CoV-2 could spread through aerosolized droplets released via patients’ exhalations.^5^ However, they noted that they cannot yet confirm whether the coronavirus identified in air samples is viable and capable of infecting through the above process.

We think that it is likely that the dosage and time of exposure would also determine whether or not infection will finally occur. Therefore, it is crucial to decide on the scenarios that will allow the transmission to longer distances. According to Pan *et al.*,^7^ experimental air sampling technologies that can detect the presence of viruses and determine their distribution in aerosol particles have many limitations and are not accurate enough, e.g., low collection efficiencies. Here, we aim at advancing the understanding of the transfer of airborne particle carriers to humans through flow modeling and simulation.

## II. MODELING

The initial modeling configuration of the problem takes into account several parameters that can influence the simulation, including the wind speed in an open environment. An accurate prediction of the transfer of airborne particle carriers to humans from a cough is governed by the following modeling considerations that must be taken into account:

1. The saliva droplet’s initial size distribution at the onset of the coughing event.
2. The human mouth-print of the cough.
3. The period of the cough and its intensity (or initial saliva droplet speed).
4. The numerical modeling approach to capture the complex varying space and time scales, e.g., both heat and mass transfer considerations, modeling of mass and phase changes due to droplet evaporation, coalescence, breakup, and turbulent dispersion in interaction with the bulk flow field.

### A. Droplet’s initial size distribution

**FIG. 1.**
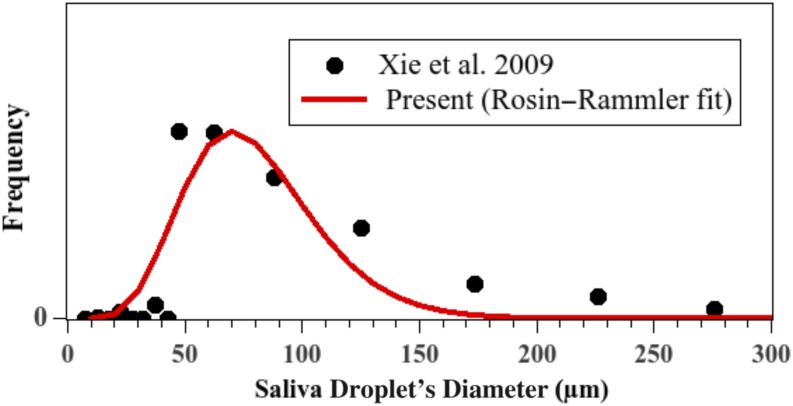
Initial saliva droplet’s size distribution. The red curve was obtained using Eq. (1). The error is approximately 6%.

Xie *et al.*^8^ conducted experimental measurements and quantified exhaled droplet’s mass and size due to talking and coughing. Moreover, they corrected the droplet’s size distribution near the origin of the ejection, which was underestimated in previous studies.^9,10^ This correction was conducted based on droplet’s dispersion analysis because larger droplets are dispersed into smaller ones gradually while moving away from the mouth jet origin. The size distribution adopted by the authors is shown in Fig. 1. It corresponds to a fit law for the data obtained by Ref. 8, fitted by the Rosin–Rammler distribution law,^11^ also known as Weibull distribution.^12^ The Weibull distribution works well for distributing cloud droplets,^13^ including water and water-like droplets. The theoretical background can be found.^14^ Figure 1 (red curve) shows the Weibull’s law of probability density function *f*. The fitting parameters are given by

$$f=\frac{n}{{\bar{d}}_{p}}{\left(\frac{d_{p}}{{\bar{d}}_{p}}\right)}^{n-1}e^{-{\left(d/d_{p}\right)}^{n}},\;n=8,\;{\bar{d}}_{p}=80\;\mu \text{m},$$ (1)

where *d*_*p*_ is the droplet diameter.

### B. Human cough mouth-print

**FIG. 2.**
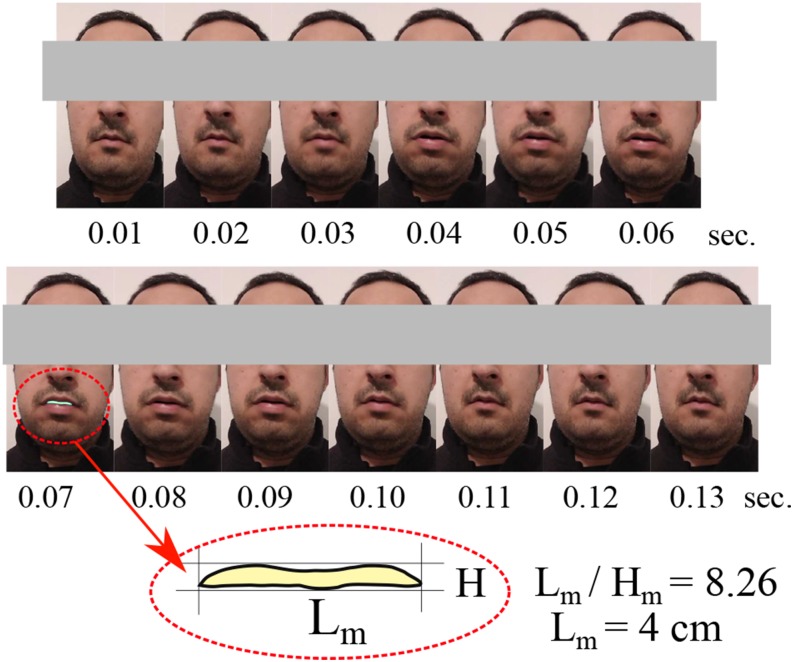
Human mouth-print during a cough period of 0.12 s captured with a high-speed camera. A rectangular sheet-like mouth-print cross section is observed at 0.07 s, corresponding to the maximum mouth opening.

During a human cough, the mouth-print can take different shapes and sizes depending on each individual’s morphology that varies from one person to another. Previous studies in the literature simplified the mouth form or shape by assigning a general hydraulic diameter.^15^ However, accurate mouth-print quantification is a critical task to capture the transport of the airborne droplet virus carriers accurately. Figure 2 illustrates an experimental measurement for a human cough captured via a high-speed camera over 0.12 s. One can observe that the maximum human mouth opening at 0.07 s has a rectangular-like mouth-print with an aspect ratio of *L*_*m*_/*H*_*m*_ = 8.26 with *L*_*m*_ ≈ 4 cm. The curved form of the mouth-print from Fig. 2 is used to create a digital mouth-print model for the saliva droplet injector in order to mimic the real droplet ejection during a human cough.

### C. Initial conditions

**FIG. 3.**
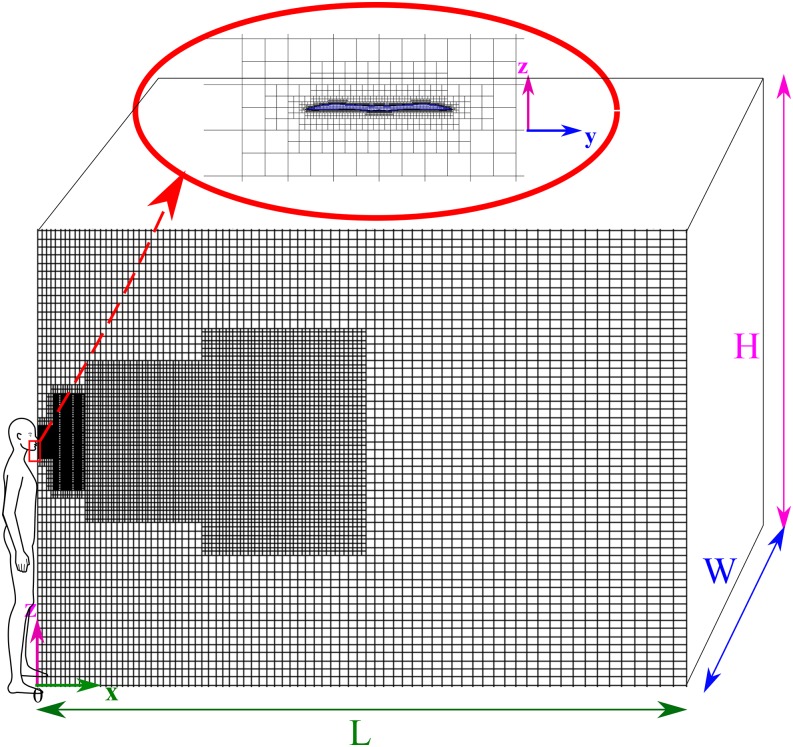
A 2D sketch of the 3D computational domain grid meshed with an advanced technique employing a hexahedral non-uniform structured mesh (≈0.5 × 10^6^ for 2 W). The mesh is very refined at the mouth-print and is gradually coarsened in the streamwise cough flow direction with multilevel refinement. Two computational domains were considered at H = 3 m, W = 1 m, and L = (4 and 6) m. The mouth-print is at z = 1.63 m.

We developed a 3D computational domain and show a 2D section in Fig. 3. We generated a mesh comprising hexahedral non-uniform structured elements or cells (≈0.5 × 10^6^). The mesh was well refined at the mouth-print and then gradually coarsened in the streamwise cough flow direction at a multilevel of refinement. The choice of this grid has been taken after conducting a grid convergence study on main local and global flow parameters, e.g., *u*_*f*_ and *p*, following a grid convergence index strategy proposed by Celik *et al.*^16^

According to van der Reijden *et al.*,^17^ saliva could have a negligible dependence on the shear rate, and its viscosity could be close to that of water. However, saliva is, in general, a complex fluid because it depends on each individual and may vary from smokers to non-smokers and diabetic people.^17^ Here, we have considered saliva to be a Newtonian fluid.

We applied a time-varying velocity inlet with particle injection at the mouth boundary to mimic the human cough over 0.12 s (Fig. 2). The velocity applied at the mouth for 0.12 s is *u*_*x*_ = 8.5 m/s, as measured by Scharfman *et al.*^15^ Using the mouth hydraulic diameter and the above velocity, the Reynolds number is *Re* = 4400. Note that if the Reynolds number is recalculated using the mouth height, it gives *Re* = 36 344, which is similar to the experimental Reynolds value of 40 000 of Scharfman *et al.*,^15^ where the flow is reported as a turbulent flow.

We applied an outlet pressure boundary condition at the outlet (y-z plane at *x* = *L*). A no-slip wall boundary condition with wall-functions for the turbulent boundary layer was applied at the ground level (x-y plane at *z* = 0). We treated the remaining boundaries as infinite domain boundaries. For non-zero wind speed cases at t > 0.12 s, we applied a constant uniform freestream velocity in the cough flow direction along the x-axis. We investigated three wind speed cases: ≈0 km/h, 4 km/h, and 15 km/h. The domain length was *L* = 4 m for wind speeds ≈0 km/h and 4 km/h. We applied a longer domain of *L* = 6 m for the highest wind speed at 15 km/h.

We considered an environment of 20 °C for the carrier fluid, 50% relative humidity, 15 °C at the ground, and 34 °C for the human mouth.

The height from the ground (at *z* = 0) to the mouth is 1.63 m corresponding to real human dimensions, with a total *H* = 3 m and *W* = 1 m. The initial total mass of the injected saliva into the domain is 7.7 mg with 1008 droplets. These values are of the same order of magnitude as those reported in the literature by Zhu *et al.*^18^ and Xie *et al.*^8^

Three different phases were initially considered inside the carrier multiphase fluid mixture: (1) dry air, (2) water vapor, and (3) liquid water. The initial mass fraction or phase-type composition of the bulk fluid is imposed as 0.991-air, 0.009-water-vapor, and 0-liquid-water. These mass fractions correspond to 50% relative humidity at ambient 20 °C and 1 atm. The mass fraction or phase-type of the droplets, ejected from the mouth, is considered as 1-liquid-water corresponding to pure liquid water-like saliva droplets.

### D. Modeling approach

For the carrier bulk multiphase fluid mixture, we have employed the compressible multiphase mixture Reynolds-averaged Navier–Stokes equations in conjunction with the *k* − *ω* turbulence model in the shear-stress-transport formulation.^19^ The governing equations are detailed in many textbooks.^20,21^

Respiratory droplets will interact with the airflow and also the ambient airflow. Droplet size and properties will influence the simulation. We know that droplets will become droplet nuclei during their dispersion and that evaporation and turbulence affect the dispersion distance. Previous studies^22^ also suggested that the size distribution and travel distances of droplet nuclei can significantly influence infection risk indoor.

Liu *et al.*^23^ showed that the droplet nuclei size, at a relative humidity of 90% (25 °C), could be 30% larger than the same droplet at a relative humidity of less than 67.3% (25 °C).

Turbulence also influences the trajectories of respiratory droplets and their wide dispersion. Liu *et al.*^23^ found that humidity influences more medium-sized droplets (60 *μ*m) than smaller and larger droplets. Larger, heavier droplets (>100 *μ*m) will leave the respiratory jet faster.

The size of droplets also varies during the evaporation and dispersion processes. Wells’^24^ classic study of airborne transmission identified the difference between disease transmission via large droplets and by airborne routes. He suggested that under normal air conditions, droplets smaller than 100 *µ*m in diameter would completely dry out before falling ∼2 m to the ground. The WHO^25^ has used Wells’ finding to establish the theory of droplets and droplet nuclei transmission depending on the size of the infecting droplet.

For the Nusselt and Sherwood numbers, we use the Ranz–Marshall model,^26,27^ which we will also use to calculate the Reynolds number modification to the quiescent evaporation rate. The subject of droplet evaporation is far from being well understood. Non-equilibrium effects become significant for initial droplet diameter less than 50 *µ*m, and the models based on the Langmuir–Knudsen law provide results in closer agreement with the experiments.^28^

Past studies have shown that detailed knowledge on the breakup of droplets is not required when applying the modified concept of a maximum stable diameter,^29^ which estimates the size of the most abundant stable fragments. Droplet acceleration is taken into account. The median mass droplet can be estimated from empirical observations that the median mass size is one-half the largest stable size particle.^29^ The above approach links together the Weber number, total breakup time, and velocity correlations for the accelerating cloud droplet. Other essential considerations concern the magnitude of the computational time step, mainly when it is significantly larger than the turbulence correlation time.^30^ Here, we use O’Rourke’s approach^30^ that involves choosing random velocity and position changes for each droplet from probability distributions that we derive for the turbulent droplet velocity and position changes.

### E. Dispersed saliva droplets phase: Two-way coupling

We treated the saliva droplets as Lagrangian particles such that each droplet is tracked individually throughout the computational domain. For each droplet, we solve differential equation, which describes the evolution of its mass, velocity, temperature, and position.

The evolution of the mass droplet is used to calculate the mass source terms of the mixture-components in the bulk carrier fluid phase and to update its pressure equation accordingly. The droplet momentum equation is used to calculate the forces exerted by the particles on the carrier phase required in the momentum equation for the fluid.

The evolution of droplet mass *m*_*p*_ (of diameter *d*_*p*_) is described by the following conservation equation:

$$\frac{dm_{p}}{dt}=-\frac{Sh}{3Sc}\frac{m_{p}}{\tau_{p}}\xi_{M},$$ (2)

in which *t* is time. *Sh*, *Sc*, $\tau_{p}=\rho_{p}d_{p}^{2}/\left(18\;\mu \right)$, and *ξ*_*M*_ are the Sherwood number, the Schmidt number, the particle relaxation time, and the potential function driving the evaporation, respectively; *ρ*_*p*_ is the particle’s density and *μ* is the dynamic viscosity of the carrier phase. The Sherwood number describes the ratio of the convective mass transfer to the mass transfer due to diffusion. The Schmidt number represents the ratio between viscous and mass diffusion rates.

As mentioned above, the heat transfer droplet model by Ranz and Marshall^26,27^ was applied to the evolution of the mass of a single saliva liquid droplet due to evaporation. It describes the heat transfer coefficient as an empirically derived correlation as a function of the Nusselt number *Nu* with the diameter droplet *d*_*p*_. Moreover, the liquid droplet breakup model by Pilch and Erdman^29^ was applied to predict the droplet’s size, and thus, the acceleration induced by the breakup depends on the Weber number *We*. The latter describes the ratio between the carrier fluid inertia forces and the droplet’s surface tension forces.

The evolution of the droplet’s velocity is computed by applying Newton’s second law of motion,

$$m_{p}\frac{du_{p}}{dt}=\sum F_{p}\left(u_{p},u_{f},B\right),$$ (3)

where *u*_*p*_ is the droplet’s velocity and *F*_*p*_(*u*_*p*_, *u*_*f*_) are the forces acting on the droplet (as a function of the droplet velocity *u*_*p*_ and also the carrier fluid velocity *u*_*f*_ interpolated at the droplet position). *B* represents the external force of gravity.

The evolution of the droplet’s temperature is obtained by solving the following energy equation based on the enthalpy difference *H*_*p*_:

$$\frac{dH_{p}}{dt}=A_{p}\left({\dot{q}}_{conv.}+{\dot{q}}_{abs.}-{\dot{q}}_{emm.}\right),$$ (4)

where *A*_*p*_ is the droplet’s surface area. From the above energy equation, *H*_*p*_ evolves over time and is the sum of heat transfer due to convection *q*_*conv*._ and radiation *q*_*abs*._ (gained from the surrounding to the particle), minus the heat transfer emitted as radiation *q*_*emm*._ or losses. The enthalpy equation can be expressed as a function of the particle temperature *T*_*p*_ such that

$$\frac{dH_{p}}{dt}=m_{p}c_{p}\frac{dT_{p}}{dt},$$ (5)

where *c*_*p*_ is the droplet’s specific heat capacity. Note that all thermophysical properties (density, heat capacity, viscosity, etc.), for both the carrier fluid and the droplets phases, are temperature-dependent. The carrier fluid is modeled as an ideal gas for its equation of state, and its transport is modeled using Sutherland’s law^31^ for its viscosity based on the kinetic theory of gases, which is suitable for non-reacting gases.

The open-source Computational Fluid Dynamics (CFD) code “OpenFOAM”^32^ was employed to solve all partial differential equations. We have used the finite volume method^33^ to discretize the carrier fluid phase. We applied second-order schemes for both time and space operators. The droplet’s Lagrangian phase equations were discretized employing semi-implicit numerical schemes at second order. The total computation time of a single case was about 1.5 days, run in parallel over 32 Intel-Xeon processors of 3 GHz frequency.

## III. RESULTS AND DISCUSSION

### A. Saliva droplets from a cough

**FIG. 4.**
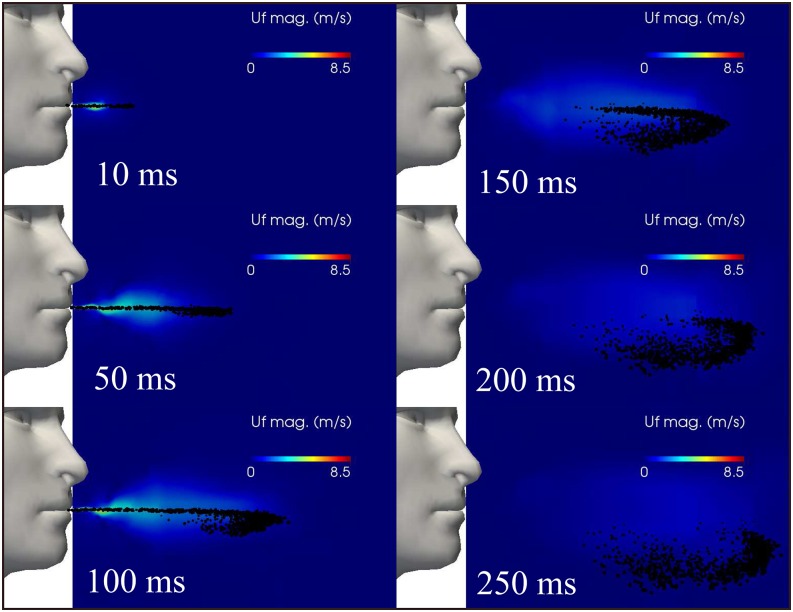
Saliva droplet cloud kinematics and dispersion show the carrier fluid flow velocity magnitude from a human cough. Wind speed ≈ 0. The total mass of ejected saliva is 7.7 mg, with 1008 total number of droplets. The environment is at ambient temperature, pressure, and relative humidity of 20 °C, 1 atm, and 50%, respectively, with the ground temperature at 15 °C and mouth temperature at 34°. The saliva droplets reach a horizontal distance of 30 cm from the mouth at t = 250 ms.

The predicted saliva droplet kinematics at the early period of ejection from a human cough are illustrated in Fig. 4 from 10 ms to 250 ms. We observe that during the applied ejection period of 120 ms (Fig. 2), the carrier fluid flow is at the maximum velocity of 8.5 m/s, which drops down gradually after closure of the mouth. A linear jet profile occurs near the mouth, which then breaks down slowly away from the mouth. In this short time at t > 120 ms, a cloud of saliva droplets is entertained inside (or carried by) the carrier fluid cloud for a short period after closure, which can be explained by the retained momentum of the droplet. At longer times, the cloud settles gradually at different rates accompanied by both dispersion and evaporation. At 250 ms, the shape of the cloud and the 30 cm maximum distance found for a droplet (horizontally away from the mouth) are of similar order of magnitude compared to previous results.^34^

**FIG. 5.**
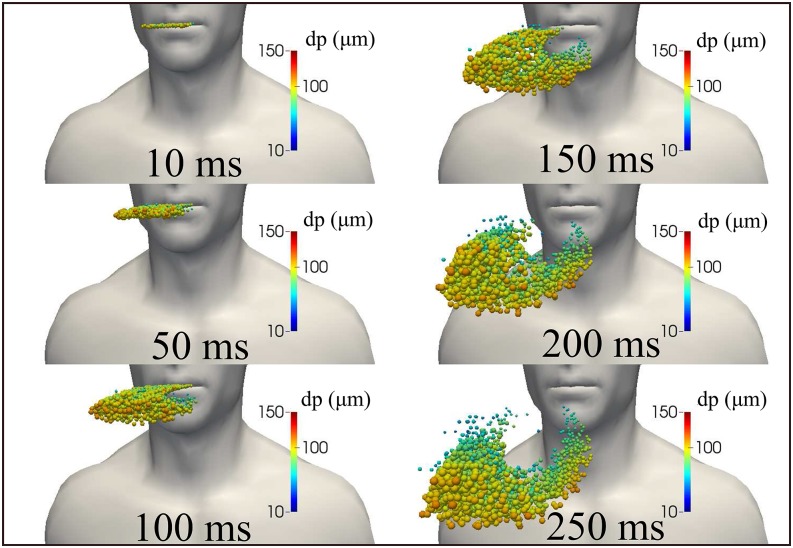
Saliva droplet cloud kinematics show the diameter of the droplets resulting from a human cough. Larger droplets settle more rapidly than smaller ones due to gravitational forces. Wind speed ≈ 0. The total mass of ejected saliva is 7.7 mg, with 1008 total number of droplets. The environment is at ambient temperature, pressure, and relative humidity of 20 °C, 1 atm, and 50%, respectively, with the ground temperature at 15 °C.

**FIG. 6.**
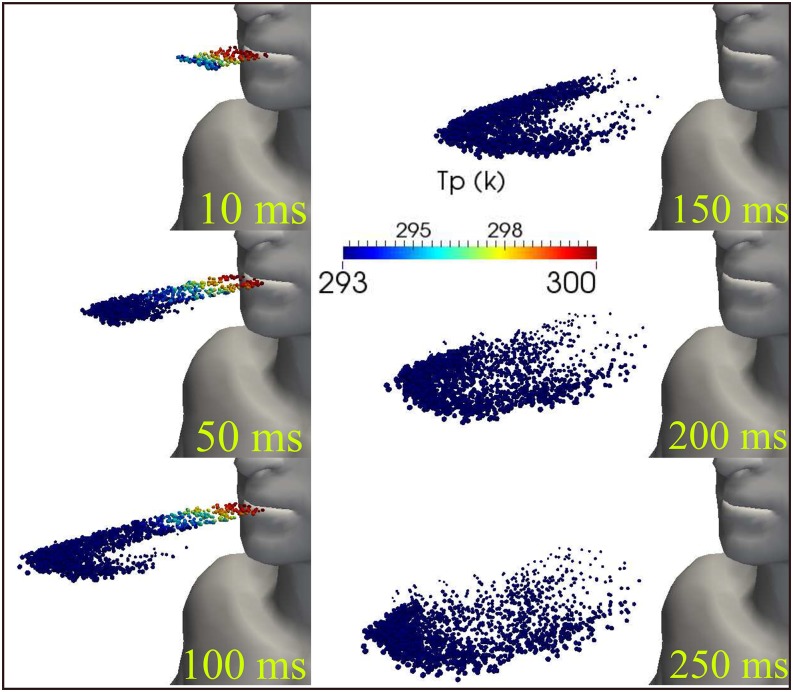
Saliva droplet cloud kinematics show the diameter droplet resulting from a human cough. Larger droplets settle more rapidly than smaller ones due to gravity. Wind speed ≈ =0. The total mass of ejected saliva is 7.7 mg, with 1008 total number of droplets. The environment is at ambient temperature, pressure, and relative humidity of 20 °C, 1 atm, and 50% with the ground temperature at 15 °C.

During a human cough, Fig. 5 shows the kinematics of the saliva droplets between 10 ms and 250 ms accompanied by droplet sizes between ≈10 *μ*m and 120 *μ*m. The temperature saliva droplet is illustrated in Fig. 6, showing hot droplets near the mouth that are cooled to lower temperature away from the mouth. This is due to a lower temperature of the surroundings at 20 °C.

### B. Airborne saliva droplet’s transport at different conditions

**FIG. 7.**
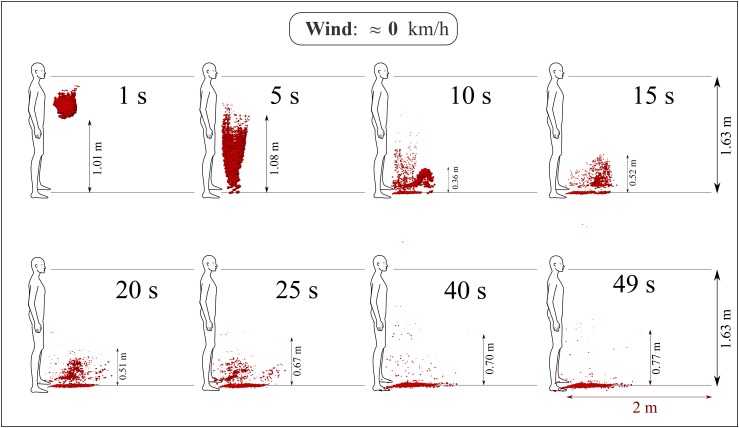
A human cough: saliva droplet’s disease-carrier particles cannot travel more than 2 m in space at approximately zero wind speed. The environment is at ambient temperature, pressure, and relative humidity of 20 °C, 1 atm, and 50%, respectively, with the ground temperature at 15 °C and mouth temperature at 34 °C.

According to several governments, strict recommendations were made for people to keep a distance of at least 6 feet (2 m). The above advice was announced to the public as a safe social distancing to prevent airborne disease transmission (such as COVID-19) from one person to another. This study shows 2 m is a safe approximate distance in the case where there is no wind, i.e., at wind speed ≈0 km/h, at 20 °C, relative humidity of 50%, and a ground surface temperature of 15 °C (Fig. 7). The ground surface temperature (GST) of 15 °C is somehow arbitrary because, in winter/spring season, the ground surface temperature is lower than the air temperature and the opposite in the summer/autumn season. Therefore, we considered GST = *T*_*air*_ − 5 °C. However, the 5 °C may vary from region to region and also depends on the soil properties. We aimed to approximate as much as possible a real situation in winter/spring seasons. Further investigation is required to quantify the effects of GST,^35^ as well as relative humidity and ambient air temperature.

Figure 7 shows the evolution of human saliva droplets, taking into account the dispersion, evaporation, breakup, and droplet settling. After 49 s, all droplets did not exceed a horizontal distance of 1 m away from the mouth. At the time of 49 s, some droplets appear at 0.77 m above the ground. At the time of 10 s, one can witness the circulation of the droplet cloud, which can be explained by its closeness to the body that plays the role of a stationary wall of no-slip like the ground surface. Also critical is that the droplets take about 15 s to fall below the human waist level, which is considered as a safe vertical distance. In the case of no wind, young children will be most vulnerable in the close vicinity of the falling droplet cloud.

**FIG. 8.**
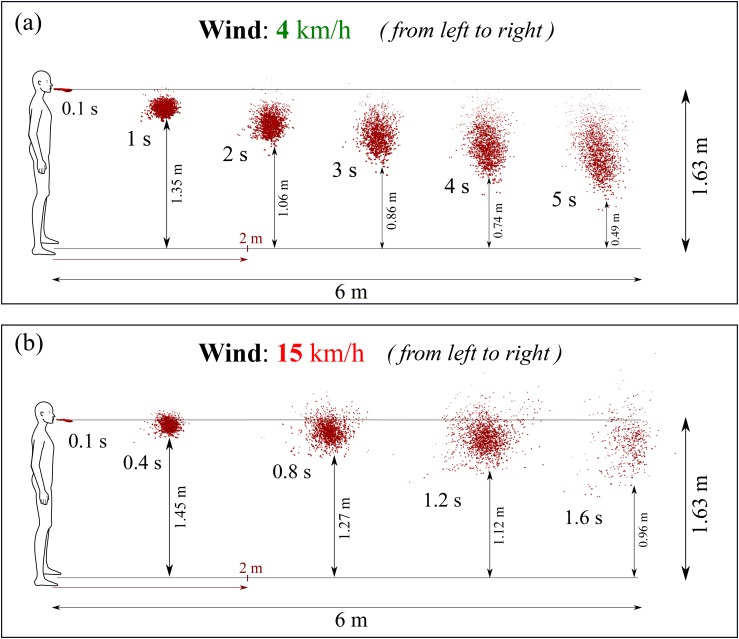
A human cough: saliva droplet’s disease-carrier particles may travel in the air medium to unexpected considerable distances depending on the environmental conditions. This figure shows the effect of wind speed on the saliva droplet and transport under dispersion and evaporation. Wind blowing from left to right at speeds of 4 km/h (a) and 15 km/h (b). The environment is at ambient temperature, pressure, and relative humidity of 20 °C, 1 atm, and 50%, respectively, with the ground temperature at 15 °C.

At 4 km/h wind speed blowing from left to right in the direction of the human cough [see Fig. 8(a)], the saliva liquid droplets can travel up to 6 m away from the mouth in a period of 5 s. The saliva droplets fly as a cloud of droplets sheared by the wind, which causes the cloud deformation under the turbulent dispersion forces. Complex phase change and transport phenomena such as evaporation and droplet breakup occur at different rates depending on the environmental conditions and on the intensity of the cough. After 5 s from the occurrence of cough, the droplet cloud loses mass, and minimum size reduces progressively until total disappearance at a critical time >5 s. Figure 8(a) enlightens another interesting phenomenon, which is the vertical stretching of the droplet cloud while moving away from the mouth where some droplets nearly reach the ground at about t = 5 s. Moreover, at this low wind speed, we observe that the saliva droplet cloud remained below the horizontal line situated 1.63 m below the mouth.

At the same environmental conditions, but with the wind speed increasing from ≈4 km/h to ≈15 km/h, we observe a different saliva droplet kinematics [Fig. 8(b)]. Under this wind speed of 15 km/h, the saliva droplets move away faster and reach 6 m in 1.6 s with an accelerating dispersion rate. Similarly, evaporation is accompanied by mass reduction in the saliva droplets, which we will discuss quantitatively in Sec. III C. Additionally, at a speed of 15 km/h, we observe that the droplet cloud is sheared and stretched along an axis, making an angle of about 45° with the horizontal line situated at 1.63 m height. The results for 15 km/h reveal that saliva droplets exist above 1.63 m height due to dispersion for all times between approximately 0.4 s and 1.6 s. The droplet cloud [Figs. 8(a) and 8(b)] will affect both adults and children of different heights. The 1.63 m assumption leaves shorter adults and children at even higher risk.

**FIG. 9.**
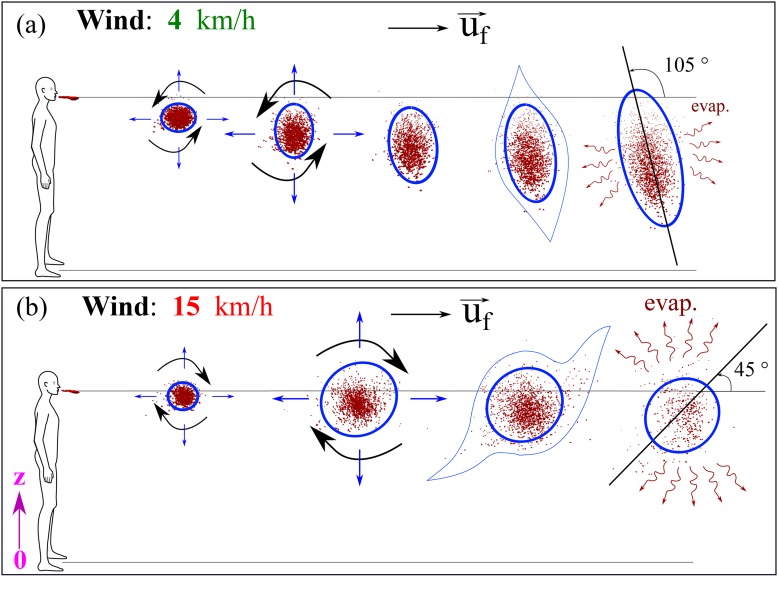
A human cough: mechanisms of airborne saliva droplet’s transport, breakup, dispersion, and evaporation. This figure shows different cloud kinematics (elongation and rotation) depending on the wind shearing force; the gravitational or settling forces; and the evaporation rates. Wind blowing from left to right at speeds of (a) 4 km/h and (b) 15 km/h. The environment is at ambient temperature, pressure, and relative humidity of 20 °C, 1 atm, and 50%, respectively, with the ground temperature at 15 °C.

We have also examined the kinematics of airborne disease-carrier saliva droplets (Fig. 9). Different saliva droplet cloud kinematics may occur at different rates such as elongation, drifting, and rotation. The cloud kinematics is very complex and has several driving forces, which are the wind shearing rate, gravitational acceleration, turbulent dispersion, interaction forces manifested by breakup or coalescence, and stress forces manifested by a droplet’s phase change or evaporation. At a low wind speed of 4 km/h [Fig. 9(a)], the saliva droplet cloud is advected in the wind direction with an increase in **anticlockwise** rotation between 0.1 s and 5 s. However, at a higher wind speed of 15 km/h [Fig. 9(b)], the saliva droplet cloud is advected in the wind direction with an increase in **clockwise** rotation between 0.1 s and 1.6 s and a 45° angle with the horizontal line at z = 1.63. The above transport evolution is explained by a reversal of the competition between some of the force ratios, e.g., wind shearing, dispersion, and settling forces. A detailed study of droplet kinematics is underway but is beyond the scope of the present study.

### C. Quantitative analysis

**FIG. 10.**
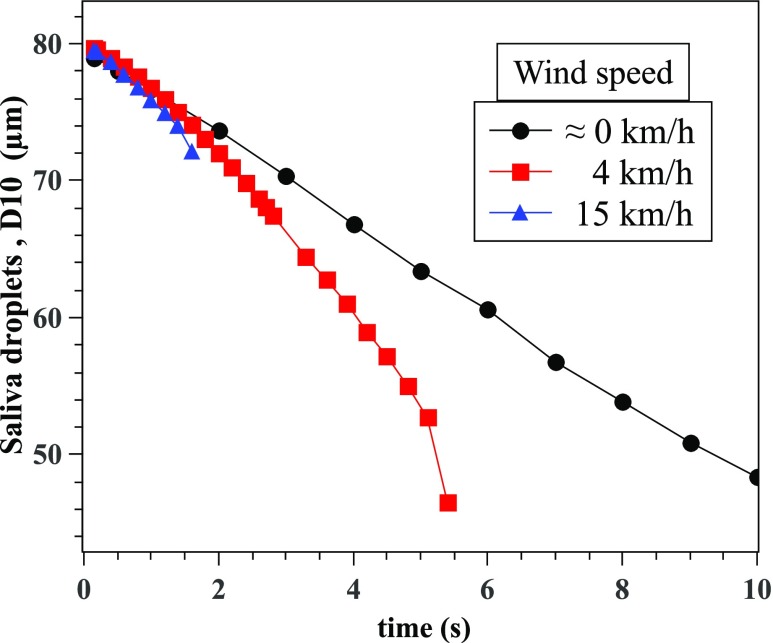
Variation of saliva droplet diameter, which represents 10% of droplets being smaller than their corresponding initial size.

We have examined the saliva droplet diameter, which represents 10% of droplets being smaller than their corresponding initial size, *D*10 in Fig. 10. For all environmental conditions including different wind speeds, the *D*10 saliva droplet diameter decreases with time but at different rates with all values varying between 45 *μ*m and 79 *µ*m. As the wind speed increases from ≈0 km/h to 15 km/h, faster and smaller *D*10 occurs. Of course, at higher wind speeds, the curve of *D*10 might disappear at a certain time because the droplet cloud has reached the outlet of the existing computational domain that is 6 m long along the cough flow direction. The evaporation process mainly causes the reduction in the *D*10 saliva droplet diameter but is also accompanied by droplet breakup and coalescence that may occur at different rates and as a function of the wind shearing intensity and the turbulent dispersion force.

**FIG. 11.**
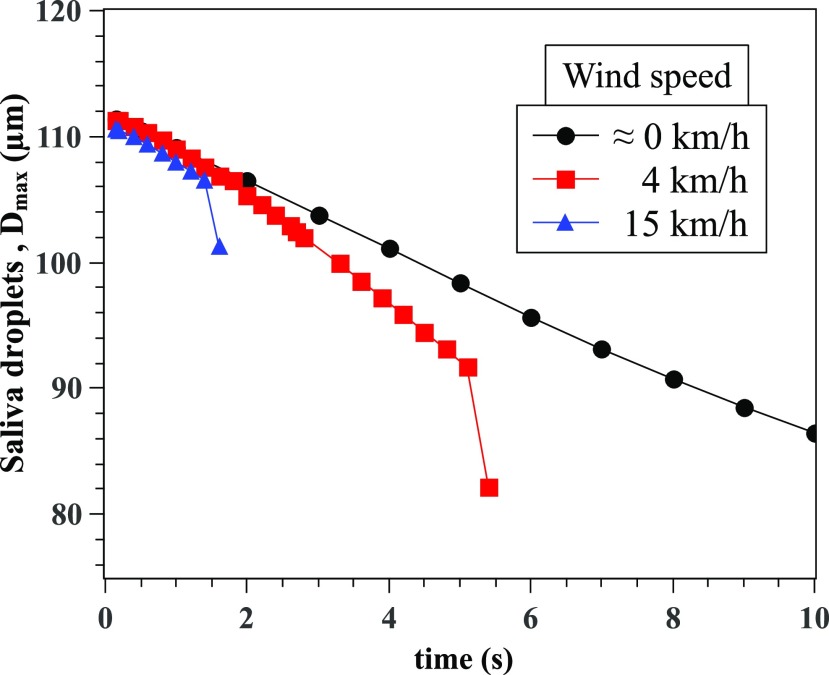
Variation of the maximum saliva droplet diameter, *Dp*_*max*_, with time.

Nevertheless, the quantification of *D*10 does not constitute a critical parameter in terms of airborne virus disease transmission compared to the maximum saliva droplet size. Bigger droplets may carry smaller virus particles and thus constitute more danger or risk in terms of airborne disease transmission between humans. Thus, the maximum saliva droplet diameter was quantified and plotted in Fig. 11 as a function of time. The maximum saliva droplet diameter *D*_*max*_ decreased with time from 111 *μ*m to 82 *µ*m at different rates. As the wind speed increases, the *D*_*max*_ reduction is observed to be faster. The latter effect is due to the higher shear rate of the wind, which accelerates droplet evaporation.

**FIG. 12.**
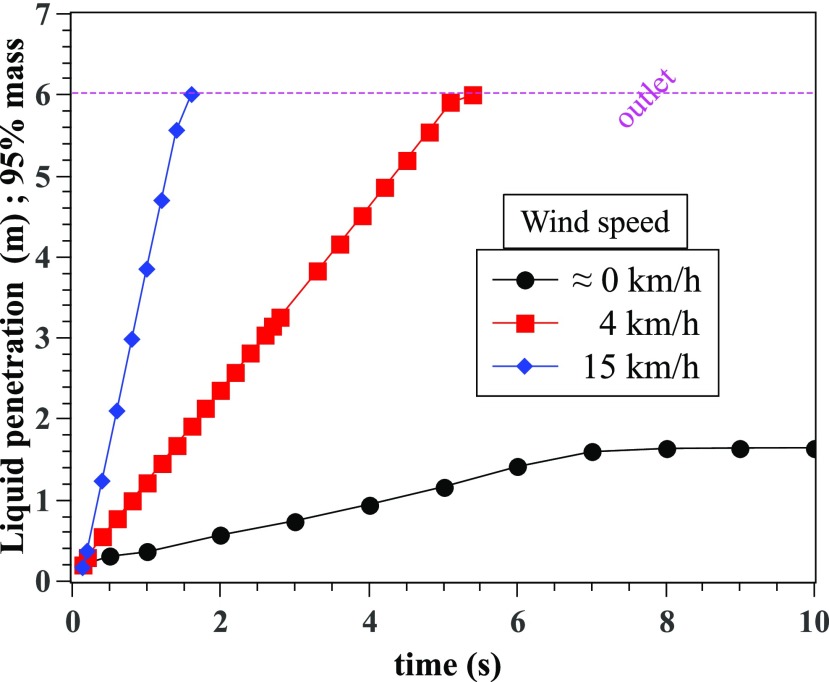
Liquid penetration distance: maximum distance traveled by a saliva liquid droplet made of 95% initial mass.

Another important quantifying factor is the liquid penetration distance (Fig. 12). It describes the maximum distance traveled by a saliva liquid droplet made of 95% initial mass. From 0 s to 10 s, at a wind speed of 0 km/h, the saliva droplets do not exceed the safe social distancing of 2 m. However, at higher speeds of 4 km/h and 15 km/h, the droplet penetration distance reaches 6 m in about 5.4 s and 1.6 s, respectively.

**FIG. 13.**
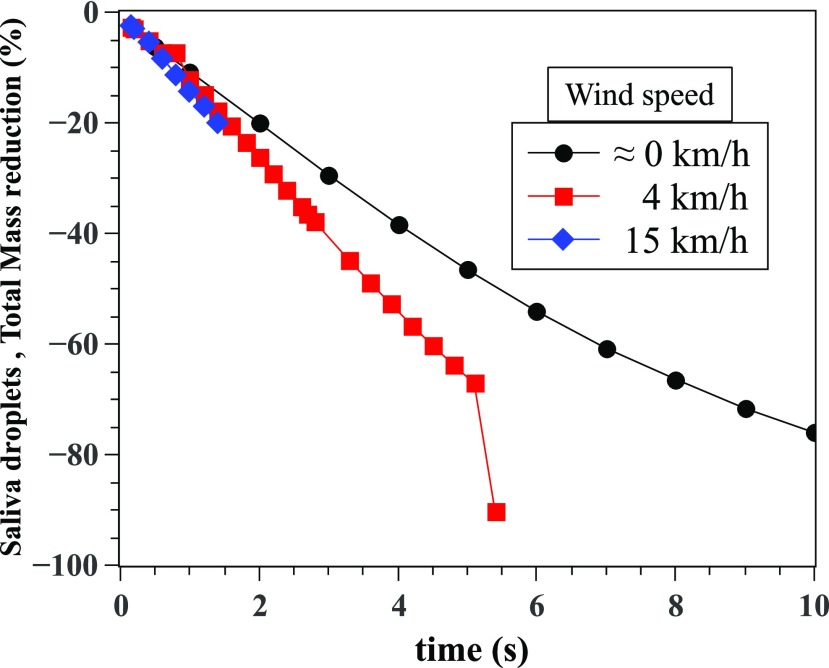
Saliva droplet’s mass reduction with reference to the initial mass.

We have also examined the total percentage of saliva droplet’s mass reduction with reference to the initial mass of 7.7 mg saliva ejected from the human cough (Fig. 13). At 4 km/h, the total mass reduction occurs more slowly than the case of 15 km/h. This finding indicates that at moderate wind speed, exposure to the droplet cloud can be longer, thus potentially increasing virus transmission risk. In Figs. 11 and 13, the last points are dropping from the distributions because the droplets have approached the outlet, which is the limit of the computational domain at 6 m. Similar data trends are observed in the literature when investigating evaporation of water droplets.^36^

## IV. CONCLUSIONS AND RECOMMENDATIONS

This study shows that, when a person coughs, the wind speed in an open space environment significantly influences the distance that airborne disease-carrier droplets travel.

1. Without the surrounding wind speed, the droplets will fall to the ground in a short distance from the person exhaling or coughing. The present analysis shows that the range may not exceed 1 m. A tiny number of particles may travel slightly further longer. Still, their trajectory beyond 1 m will already be at a height significantly below half a meter dropping toward the ground. Thus, these droplets may not constitute a risk regarding facial contact of adults at this distance.
2. At wind speeds from 4 km/h to 15 km/h, we found that saliva droplets can travel to distances up to 6 m with a decrease in concentrations and liquid droplet size in the wind direction. Our findings imply that depending on the environmental conditions, the 2 m social distance may not suffice. Further research is required to quantify the influence of other parameters such as the environment relative humidity and temperature among others.
3. The droplet cloud will affect both adults and children of different heights. Shorter adults and children could be at higher risk if they are located within the trajectory of falling droplets.
4. At a lower wind speed, the total mass reduction occurs more slowly compared to a higher speed, which may prolong the exposure of a human to the droplets if the subject is located within the droplet’s envelope.

Overall, the results show that in open spaces, airborne droplet carriers can travel significantly further than the 2 m recommended distance due to the wind speed. Several areas need further investigation to examine the impact of the above findings:

• A recent letter discussed the COVID-19 outbreak associated with air conditioning in a restaurant in Guangzhou, China.^37^ Therefore, it would be worth mentioning generalizing the current analysis to an indoor setting.
• We need to understand the droplet evaporation more deeply, especially at different environmental conditions.
• We should also carry out further research to determine the droplet size at the origin. Droplet evaporation depends on the time it takes for the droplet to travel from the mouth to a particular position.
• The violent cough of patients with respiratory diseases will affect droplet generation and secretions of fluids on airway surfaces and heighten coughing frequency.^38^ These factors need to be further quantified.
• Further research is also required to assess the probability of viral transmission vs droplet. This study shows that the droplet concentration can be significant up to considerable distances from the origin of the cough.

The issues arising from the past and the recent pandemic require a holistic approach to elucidate the open scientific questions and address the practical challenges. Such an approach would require closer interaction between bio-medicine, engineering fluid physics, and social sciences.

## SUPPLEMENTARY MATERIAL

See the supplementary material for the airborne droplet transmission at different wind speeds.

## DATA AVAILABILITY

The data that support the findings of this study are available on request from the authors.
